# Dog–Owner Relationship, Owner Interpretations and Dog Personality Are Connected with the Emotional Reactivity of Dogs

**DOI:** 10.3390/ani12111338

**Published:** 2022-05-24

**Authors:** Sanni Somppi, Heini Törnqvist, Aija Koskela, Antti Vehkaoja, Katriina Tiira, Heli Väätäjä, Veikko Surakka, Outi Vainio, Miiamaaria V. Kujala

**Affiliations:** 1 Department of Equine and Small Animal Medicine, Faculty of Veterinary Medicine, University of Helsinki, P.O. Box 57, FI-00014 Helsinki, Finland; heini.tornqvist@gmail.com (H.T.); aija.koskela@helsinki.fi (A.K.); katriina@smartdog.fi (K.T.); outi.vainio@helsinki.fi (O.V.); miiamaaria.v.kujala@jyu.fi (M.V.K.); 2 Department of Psychology, Faculty of Education and Psychology, University of Jyväskylä, P.O. Box 35, FI-40014 Jyväskylä, Finland; 3 Faculty of Medicine and Health Technology, Tampere University, P.O. Box 692, FI-33101 Tampere, Finland; antti.vehkaoja@tuni.fi; 4 Research Group for Emotions, Sociality, and Computing, Faculty of Information Technology and Communication Sciences, Tampere University, P.O. Box 100, FI-33014 Tampere, Finland; heli.vaataja@lapinamk.fi; 5 Master School, Lapland University of Applied Sciences, Jokiväylä 11 B, FI-96300 Rovaniemi, Finland; veikko.surakka@tuni.fi; 6 Department of Neuroscience and Biomedical Engineering, Aalto University School of Science, P.O. Box 12200, FI-00076 Aalto, Finland

**Keywords:** *Canis familiaris*, behavior, heart rate variability, autonomic nervous system, emotions, human–animal interaction

## Abstract

**Simple Summary:**

The relationship between owner and the dog affects the dog’s attachment behaviors and stress coping. In turn, the quality of the relationship may affect owner’s interpretations about their dog’s behavior. Here, we assessed dogs’ emotional responses from heart rate variability and behavioral changes during five different situations. Dog owners evaluated the emotion (valence and arousal) of their dog after each situation. We found that both negative and positive incidents provoked signs of emotional arousal in dogs. Owners detected the dog’s arousal especially during fear- and stress-evoking situations. The dog–owner relationship did not affect owners’ interpretation of dogs’ emotion. However, the dog–owner relationship was reflected in the dog’s emotional reactions. Close emotional bond with the owner appeared to decrease the arousal of the dogs. Dog owners’ frequent caregiving of their dog was associated with increased attachment behaviors and heightened arousal of dogs. Owners rated the disadvantages of the dog relationship higher for the dogs that were less owner-oriented and less arousable. Dog’s arousal may provoke dog’s need to seek human attention, which in turn may promote the development of emotional bond.

**Abstract:**

We evaluated the effect of the dog–owner relationship on dogs’ emotional reactivity, quantified with heart rate variability (HRV), behavioral changes, physical activity and dog owner interpretations. Twenty nine adult dogs encountered five different emotional situations (i.e., stroking, a feeding toy, separation from the owner, reunion with the owner, a sudden appearance of a novel object). The results showed that both negative and positive situations provoked signs of heightened arousal in dogs. During negative situations, owners’ ratings about the heightened emotional arousal correlated with lower HRV, higher physical activity and more behaviors that typically index arousal and fear. The three factors of The Monash Dog–Owner Relationship Scale (MDORS) were reflected in the dogs’ heart rate variability and behaviors: the Emotional Closeness factor was related to increased HRV (*p* = 0.009), suggesting this aspect is associated with the secure base effect, and the Shared Activities factor showed a trend toward lower HRV (*p* = 0.067) along with more owner-directed behaviors reflecting attachment related arousal. In contrast, the Perceived Costs factor was related to higher HRV (*p* = 0.009) along with less fear and less owner-directed behaviors, which may reflect the dog’s more independent personality. In conclusion, dogs’ emotional reactivity and the dog–owner relationship modulate each other, depending on the aspect of the relationship and dogs’ individual responsivity.

## 1. Introduction

Currently, it is widely recognized that dogs have basic emotions and affective states, although their experience of emotion is not directly measurable (for reviews, see [1,2]). Canine emotions have most commonly been assessed based on observation of dogs’ behavior. Affective states can be reflected in dogs’ facial expressions, vocalizations, postures and movement of the whole body and, more specifically, ears, eyes, mouth and tail [3,4,5]. However, emotion-related signals can be very subtle, and a single cue alone does not necessarily indicate a certain emotion. For example, mouth licking and tail wagging has been associated with both positive and negative emotional situations [4,6,7]. Observable emotion-related behavior may also vary among individuals [5].

Studying dog behavior together with physiology can provide a more thorough understanding of dog emotional reactivity than either of the two alone [5]. Recently, the dogs’ emotion-related responding has been studied also with physiological measures from autonomic nervous system. Autonomic nervous system has two branches: sympathetic and parasympathetic. Sympathetic activity increases physiological and affective readiness, allowing for fast reaction, while parasympathetic activity decreases excitation. The alterations between these two systems result in beat-to-beat heart rate variation, which can be measured as heart rate variability (HRV) [8,9]. The decrease in HRV reflects reduced vagal tone and a dominance of the sympathetic nervous system on cardiac activity during increased physical efforts and/or emotional arousal [8]. Notably, emotional arousal can increase due to both positive and negative experiences. For example, in dogs, positive anticipation and rewarding [10,11,12,13] as well as discomfort, anxiety and fear [14,15,16,17] have been associated with a decrease in HRV. Inversely, an increase in HRV has been linked to a more relaxed affective state in dogs [16,18,19,20,21]. Furthermore, as evidenced in humans [22], the personality type can be reflected in the HRV in dogs. For example, anxiety and aggression-related traits have been linked to lower HRV in dogs [23,24,25]. 

Changes in affective states may alter dogs’ motor activity, for example, physical activity increases due to distress [26,27,28] and positive arousal [29,30]. Thus, physical activity may be used as an aid in measuring emotional behaviors. Physical motion may complicate the interpretation of HRV, as cardiac activity is affected by both emotional and physical reactivity [8,9]. Increased motion modulates HRV because of the changes in energy metabolism [31,32] and interferes the measurement due to muscle movement-generated artifacts [33,34,35]. Thus, physical activity should be considered, especially in studies where HRV is used to assess physiological responses of affective states in freely moving animals [8,32]. However, only a few emotion-related HRV studies in dogs report the effect of activity [10,16,36]. With the current, wide availability of activity trackers, activity can be taken into account more easily and precisely in the experiments of canine emotions.

When the canine emotions are set to a wider context, the dog owner and the dog–owner relationship plays a big role in the lives of pet dogs. Sharing the everyday life and close interaction with the dog gives owners a possibility to detect changes in the emotional states of their dogs. Owners’ perceptions of their dogs’ emotions have been often assessed with ratings of discrete emotions, such as fear, happiness and aggressiveness [37,38,39,40]. However, the dimensional theory of emotional approach—which conceptualizes affective states along valence and arousal dimensions [41,42]—may be useful [43], especially for exploring physiological correlates of canine emotions [44]. When dogs’ behaviors are described freely by using affective terms, the ratings fall quite well into these emotional dimensions [37,45,46]. However, surprisingly little research exists on how well owners’ interpretations of their dogs’ affective states actually correlate with physiological measures of emotion and results have been contradictory [47,48].

In addition, as dog–owner attachment relationship may have a role in the owner’s perception of dog’s behavior and emotions [38,39,43], the dog–owner bond has a true reflection in physiology of both parties through the hormones that mediate attachment behavior and stress coping [49,50]. The social bond between pet dogs and their owners resembles the attachment between parent and child [51,52] and includes characteristics present in friendship [53]. The parent–child attachment bond can be described with four behavioral components [54] which are found also in dogs: (1) A safe haven: in a frightening situation, the owners presence alleviates dog’s stress responses [14]. (2) A secure base effect: in the presence of the owners, dogs are less hesitant to explore a new environment and acts more actively in challenging situations [55,56]. (3) Separation distress: dogs show signs of distress when isolated from the owner. (4) Proximity seeking: dogs stay close distance to owner and show attention-seeking behaviors toward the owner when they are uncertain or distressed. Proximity seeking is also related to affiliative behavior during, for example, reunion after separation [52,55]. Secure attachment, strong emotional bond and positive interactions between the dog and the owner are associated with reduced level of stress in dogs [57,58,59]. The quality of the dog–owner relationship seems to modulate the dog’s long-term stress coping [60,61]. However, the effect of the owner–dog relationship on dogs’ emotional regulation may depend also on the dog personality [61,62].

In this extensive, multi-method study, one of our main objectives was to clarify how different emotional situations affect the dogs’ physiological responses (HRV) of dogs, and whether HRV, owner evaluations and dogs’ behavior complement each other during different situations. We were also interested in how the owner–dog relationship relates to dogs’ behavior, HRV, and the owners’ interpretations. The dogs’ behavior was assessed from video recordings, HRV from the cardiac measurements, physical activity from 3D acceleration recordings and the owners’ assessments of dog valence and arousal in five emotion-provoking situations with their owners. The owner–dog relationship and the dog’s personality traits were evaluated with validated questionnaires [63,64]. We expected the following: (1) dog HRV, activity and behavior differ in different emotional situations; (2) dog owner evaluations of the dog emotional state complement the HRV results in different emotional situations; (3) dog–owner relationship affects the dog HRV; and (4) dog HRV and behavior are connected with the dog–owner relationship in a situation-dependent way.

## 2. Materials and Methods

**Subjects.** A total of 33 healthy pet dogs from two breeds (Border Collies and Labrador Retrievers) participated in the study. Due to technical failure, heart rate data could not be achieved from four dogs, and therefore a total of 29 dogs were included in the final dataset: 15 Border Collies and 14 Labrador Retrievers (12 intact females, 5 neutered females, 9 intact males, 3 neutered males), average age 4.3 years (SD 2.1 y, range 1.3–10.8 y) and average weight 25.3 kg (SD 7.5 kg, range 15.5–43.0 kg). Some of the subjects were recruited through social media (Facebook, Twitter) and some by smartDOG company, Yorkshire, UK, which offers cognitive testing for dog owners (https://smartdog.fi/english/, accessed on 25 March 2022). In the recruitment announcement, the exclusion criteria for dogs were severe separation anxiety, shyness or aggression towards strange humans, according to the owners. All the dogs lived indoors with their owners and had been actively trained for a dog sport, such as agility or obedience training, and thus the sample included competition and working dogs. Twenty-four dogs were the only dog of the owner. All the dog owners were female. Twenty-two of the owners had owned a dog before the current dog and twenty-seven owners reported that they were the main responsible person of the dog in their family. Eight of the owners had dog-related job or education.

**Figure 1:**
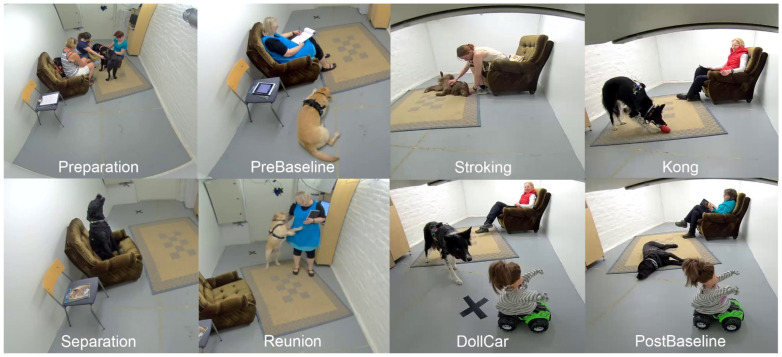
Screenshots from the test, recorded with two video cameras from opposite walls. Preparation illustrates the testing room area with the furniture. The whole test comprised seven different phases: PreBaseline, Stroking, Kong, Separation, Reunion, DollCar and PostBaseline.

**Experimental procedures.** Experiments were conducted in the Faculty of Veterinary Medicine, University of Helsinki from May to June 2018. Dogs participated in the behavior test together with their owners. The test was conducted in a windowless room (4.82 × 2.44 m), which was furnished with two chairs, a carpet and a bookshelf with a computer screen (see Figure 1).

Behavior test comprised seven phases (Figure 1 and Table 1) conducted in the same, pre-defined order for every dog. Before the test commenced, the owner was given instructions regarding the study execution, the dog was allowed to familiarize with the testing room, and the wearable data acquisition equipment were worn on the dog. After these preparations (approx. 25 min), the experimenters left the room, leaving the dog with the owner. During the test the dog was allowed to behave freely. Owners acted according to the written instructions, which appeared on the screen before each test phase. Experimenters followed the test procedure from another room via two video cameras. If necessary, experimenters gave the instructions by a radiotelephone, e.g., if the owner did not notice the written instructions. There was approx. 1–3 minute gap between the test phases, during which the owner answered the queries about the emotional state of the dogs.

**Cardiac measurements.** During the behavior test, dogs wore a neck collar and a custom-made neoprene harness with integrated Polar Soft Strap electrode belt ([65]; visible in Figure 1, phases PreBaseline and Reunion). Dogs’ heart rate was monitored with Polar RS800CX and Polar H1 heart rate sensor (Polar Electro Oy, Kempele, Finland). Conductive ultrasound transmission gel (AquaSonic100, Parker Laboratories Inc., Fairfield, CA, USA) was used to improve the ECG signal. Dogs’ fur was not shaved. Heartbeat intervals were reported with Polar sensors with 1 ms resolution. Simultaneously with the harness, dog’s electrocardiogram (ECG) was recorded as a part of another study. 

The heart rate data were visually inspected and noisy segments were omitted from further analysis. The R-R data were corrected using the inbuilt ‘artefact correction’ of Kubios software (Kubios Oy, Kuopio, Finland). The criteria for correction threshold was 450 ms, which means that if beat-to-beat interval differs more than 450 ms from the previous or the following intervals, it was corrected by Kubios algorithms. The 5 min segments with less than 15% corrected beats were included in the analysis. A total of 186 segments with average correction of 5.4% (SD 4.4%) were included in the statistical analyses. Seven segments did not reach the criteria (Stroking phase 3 segments, Separation 1 segment, Reunion 2 segments, PostBaseline 1 segment). From the corrected data, HR (beats per minute), RMSSD (square root of the mean squared differences in successive NN intervals) were calculated by Kubios software. RMSSD was chosen because it has been shown to be reliable for measuring HRV in dogs with Polar^®^ heart rate meters [66,67] and it is interfered by motion-related artifacts less than other commonly used HRV parameters [32]. No gold standard exists on which HRV parameters should be used when studying emotion-related cardiac activity in freely moving canines. In general, some parameters used in humans (e.g., pNN50 and Poincaré plots) are not valid for dogs [68].

**Activity measurement.** A triaxial accelerometer ActiGraph GT9X Link (Actigraph LLC, Pensacola, FL, USA) was placed on the back belt of the dog’s harness. The accuracy of ActiGraph GT9X Link accelerometers is better for behavior classifications when placed on back of the dog compared to being placed in neck [69]. The activity was measured at the sampling rate of 100 Hz. From the accelerometer data of the ActiGraph GT9X Link, the minute-by-minute total activity value was extracted as three axial vector magnitude (counts per minute) for 60 s epochs using ActiLife software (ActiGraph LLC, USA).

**Questionnaires about dog personality and dog–owner relationship.** During the Pre-Baseline phase of the behavior test, dog owners filled in paper versions of commonly used validated questionnaires assessing dogs’ personality, behavior and dog–owner relationship, translated to Finnish: (1) Monash Canine Personality Questionnaire Revised “MCPQ-R” [64], likert scale 1–5 with the following factors: Self-assuredness (MCPQR-S); Neuroticism (MCPQR-N); Amicability (MCPQR-A); Trainability (MCPQR-T); Extraversion (MCPQR-E); and (2) Monash Dog Owner Relationship Scale “MDORS” [63], with the following factors: Emotional Closeness (MDORS-EC), Perceived costs (MDORS-PA), Shared activities (MDORS-SA). MDORS uses a likert scale 1–5 for EC and PC and a frequency scale 1–5 for SA. The owners were instructed to consider their dog in general when answering the questionnaires, not only related to the testing situation. The reliability of questionnaire data were checked by calculating Cronbach α (Cr-α) for each factor. Factors with Cr-α < 0.6 were corrected by omitting the invalid items (Table 2). Average values for each MCPQ-R and MDORS factors were calculated after the corrections.

**Owner ratings of dog’s emotional state.** After the each test phase of the behavior test owners evaluated the emotional valence and arousal of their dogs by using iPad Pro A1709 (Apple Inc., Cupertino, CA, USA) experience sampling application (RealLifeExp, LifeData^®^, Marion, OH, USA). The valence was asked in Finnish “How pleasant your dog’s emotional state is now” and arousal “How aroused your dog is now?”. Owners answered on a visual analogue scale from 0 to 100: for valence, 0 = very negative, i.e., unpleasant, 100 = very positive, i.e., pleasant; for arousal, 0 = very low, i.e., calm, 100 = very high, i.e., excited). Owners were instructed to answer according to their own subjective impression. During the separation phase owners observed their dog through the video camera.

**Behavioral recordings.** Behavior test was video recorded with two wide-eye video cameras (D-Link DCS-2530L, D-Link ltd., Taipei, Taiwan) positioned in opposite walls of the testing room. The duration in seconds and frequency of a total of sixteen discrete behaviors were annotated from video recordings by a biologist specialized with ethology with Observer XT 10.5 (Noldus, Wageningen, The Netherlands). The annotated behaviors are described Table 3. The sum variables (Fear behaviors; Self-directed behaviors) which have been typically categorized as signs of fear and stress in dogs [15,26,70,71].

**Statistical analysis.** The general differences between test phases in physical activity, heart rate variability and owners’ assessments of dogs’ emotional state were analyzed with non-parametric Friedman test and Wilcoxon signed rank test with statistical analysis software SPSS 25.0 (IBM, New York, NY, USA). 

The effects of the dog–owner relationship (MDORS factors) and dogs’ personality (MCPQ-R factors) on the heart rate variability were analyzed with generalized linear mixed models (GENLINMIXED), using normal distribution and identity link function with variance components (VC) covariance structure. The model selection was based on the evaluation of Akaike Information Criteria, linearity of observed-by-predicted plots and normality of Pearson residual plots. The factor selection was performed by stepwise backward procedure. The fixed factors included in the final model were Phase (Pre-Baseline, Stroking, Kong, Separation, Reunion, Post-Baseline), breed, MDORS-EC, MDORS-SA and MDORS-PC. The mean score of physical activity and the age of the dog were included as covariates. The personality factors (MCPQR-S, MCPQR-N, MCPQR-A, MCPQR-T and MCPQR-E) and sex were omitted from the final model because they did not reach statistical significancy.

In the post hoc tests, *p*-values were corrected with sequential Bonferroni adjustment. The results are reported as estimated means with standard error of mean (SEM) or 95% confidence interval (CI) using the significance level *p* < 0.05 for the corrected values. The heart rate parameters were square root transformed for analysis to acquire better model fitting, and they are reported as transformed values.

Correlations between heart rate variability, physical activity, dogs’ behaviors, owners’ emotion ratings and the aspects of dog–owner relationship were analyzed with Spearman’s Rho within each test phase. In addition, general correlation between dogs’ personality traits (MCPQ-R) and dog–owner relationship factors (MDORS) were analyzed with Spearman’s Rho.

## 3. Results

### 3.1. General Differences between the Test Phases on Dog HRV, Activity and Dog Owner Assessment of Dog Emotion

**Figure 2:**
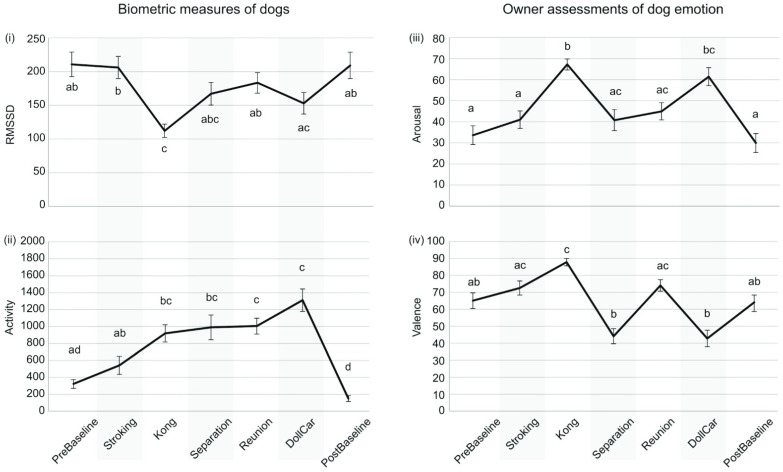
General differences between the test phases in the dog biometric measures and the owner assessments. (**i**) Dog HRV in different test phases, measured as mean RMSSD + SEM (ms). (**ii**) Dog physical activity measured as vector magnitude + SEM (counts per minute). (**iii**) Owner assessment of dog emotional arousal (mean + SEM) on a scale from 0 to 100 (0 = very low, i.e., calm, 100 = very high, i.e., very excited). (**iv**) Owner assessment of dog emotional valence (mean + SEM) on a scale from 0 to 100 (0 = very negative, i.e., unpleasant, 100 = very positive, i.e., very pleasant). Statistically significant differences (*p* < 0.05, Bonferroni-corrected) between the test phases are marked with different letters. The exact numerical statistics for comparisons between the test phases can be found in Table S1.

The mean HRVs measured as RMSSD were statistically different in the different phases of the test (*p* < 0.001, Friedman test). During the Kong phase RMSSD was lower than during the baselines, Stroking and Reunion. During DollCar phase RMSSD was also lower than in Stroking phase, but DollCar phase did not differ from other phases (Figure 2i).

The mean physical activity of the dogs differed at different phases of the test (*p* < 0.001). Activity was lowest during the Baselines and highest during the DollCar phase (Figure 2ii).

Owners’ ratings of emotional arousal were statistically different at different phases of the test (*p* < 0.001). Arousal was rated as highest during the Kong and DollCar phases (Figure 2iii). Owners’ ratings of emotional valence differed at different phases of the test (*p* < 0.001). Emotional valence was rated most positive during Kong and most negative during Separation and DollCar (Figure 2iv).

### 3.2. The Effects of Dog–Owner Relationship (MDORS) and Activity on HRV

In-depth analysis with GLMM, in which the effects of dog–owner relationship and physical activity were taken into account, RMSSD differed between the test phases (<0.001). During Kong phase RMSSD was significantly lower than during the other phases except the PostBaseline (Kong 111.69 ms, CI 95.13–129.59 ms vs. PreBaseline 174.76 ms, CI 114.80–207.54 ms, *p* = 0.008; Kong 111.69 ms, CI 95.13–129.59 ms vs. Stroking 186.77 ms, CI 157.46–218.58 ms, *p* < 0.001; Kong 111.69 ms, CI 95.13–129.59 ms vs. Separation 173.76 ms, CI 147.93–201.67 ms, *p* = 0.002; Kong 111.69 ms, CI 95.13–129.59 ms vs. Reunion 186.02 ms, CI 159.82–214.21 ms, *p* < 0.001; Kong 111.69 ms, CI 95.13–129.59 ms vs. DollCar 166.57 ms, CI 138.87–196.78 ms, *p* = 0.014; Kong 116.69 ms, CI 95.13–129.59 ms vs. PostBaseline 165.42 ms, CI 129.05–206.29 ms, *p* = 0.157). Other test phases did not differ statistically significantly from each other in RMSSD.

The factors of dog–owner relationship affected RMSSD. Higher RMSSD was associated with higher scores in Perceived Costs factor (MDORS-PC) (coefficient 1.895, SE 0.7180, *p* = 0.009) and Emotional Closeness factor (MDORS-EC) (coefficient 0.605, SE 0.2490, *p* = 0.016). Additionally, a trend showed that lower RMSSD was associated with higher scores in Shared Activities factor (MDORS-SA) (coefficient −0.945, SE 0.5134, *p* = 0.067). Breeds did not differ in RMSSD (*p* = 0.627). Age was linked to heart rate variability so that older dogs had lower RMSSD (coefficient −0.276, SE = 0.112, *p* = 0.015).

Physical activity affected RMSSD so that higher activity scores were associated with lower RMSSD (coefficient −0.002, SE = 0.0004, *p* < 0.001).

### 3.3. Correlations of Different Dog- and Owner-Related Factors

#### 3.3.1. Correlations of Dog Behaviors with HRV and Physical Activity

Dog behaviors correlated statistically significantly with dog HRV and physical activity depending on the experimental phase (Table 4; correlations between the behavior variables are reported in Table S2).

RMSSD correlated negatively with several behaviors: fear behaviors during DollCar; vocalizations during Separation and DollCar; panting during Stroking and Separation; interaction with the owner during PreBaseline; visit the owner during PreBaseline; tail wagging during Reunion; yawning during Reunion (Table 4).

Activity correlated positively with several behaviors: fear behaviors during DollCar and PostBaseline; panting during DollCar; vocalizations during Separation, DollCar and PostBaseline, interaction with the owner during PreBaseline, DollCar and PostBaseline; tail wagging during Stroking, Kong and DollCar; yawning during PostBaseline; self-directed behaviors during PreBaseline and PostBaseline (Table 4).

#### 3.3.2. Correlations of Owners’ Ratings of Dog Emotional State with Dog HRV, Activity and Behaviors

Owners’ arousal ratings correlated negatively with owners’ valence ratings during during PreBaseline (r = −0.558, *p* = 0.002), PostBaseline (r = −0.650, *p* < 0.001), Separation phase (r = −0.690, *p* < 0.001) and DollCar phase (r = −0.611, *p* < 0.001). During these situations, the more negative owners rated the dogs’ emotional state, the higher they rated dogs’ arousal. During positive situations (i.e., Stroking, Kong, Reunion) owners’ arousal and valence ratings were not correlated.

**Owners’ ratings of dog emotional arousal.** Owners’ arousal ratings correlated negatively with RMSSD and positively with physical activity during negative phases (i.e., Separation and DollCar. During baselines and positive phases (i.e., Stroking, Kong and Reunion) owners’ arousal ratings did not correlate with RMSSD (Table 5).

Owners’ arousal ratings correlated positively with several behaviors depending on the test phase (Table 6): fear behaviors during DollCar; vocalizations during Separation, DollCar and PostBaseline; panting during Stroking and Reunion; interaction with owner during Reunion; visit the owner during PostBaseline; tail wagging during Kong, Reunion and PostBaseline; yawning during PostBaseline; self-directed behaviors during Stroking and PostBaseline. In addition, self-directed behaviors correlated negatively with arousal ratings during Kong phase.

**Owners’ ratings of dog emotional valence.** Owners’ valence ratings correlated positively with RMSSD during Separation and DollCar and negatively with physical activity during Separation and PostBaseline. During baselines and positive phases (i.e., Stroking, Kong and Reunion) owner’s valence ratings did not correlate with RMSSD (Table 5). 

Owners’ valence ratings correlated negatively with several behaviors depending on the test phase (Table 6): fear behaviors during DollCar and PostBaseline; vocalizations during Separation and DollCar; panting during Separation; interaction with owner during PostBaseline; visit the owner during PostBaseline; close to owner during DollCar; yawning during PreBaseline and PostBaseline; self-directed behaviors during PostBaseline. In addition, owners’ valence ratings correlated positively with tail wagging during Kong and close to owner during Reunion and PostBaseline.

#### 3.3.3. Correlations of Dog–Owner Relationship (MDORS) with Dog Behaviors

**Emotional closeness factor** (MDORS-EC) was positively correlated with staying close to owner during the Kong phase (r = 0.430, *p* = 0.020), yawning during Reunion (r = 0.593, *p* = 0.001) and fear behaviors during DollCar (r = 0.400, *p* = 0.032). **Shared activities factor** (MDORS-SA) was positively correlated with interaction with the owner (duration) during PreBaseline (r = 0.386, *p* = 0.039), panting during Reunion and PostBaseline (r = 0.395, *p* = 0.041; r = 0.450, *p* = 0.021), interaction with the door during Separation (r = 0.349, *p* = 0.038), yawning during Reunion (r = 0.626, *p* < 0.001) and self-directed behaviors during Reunion (r = 0.492, *p* = 0.009). **Perceived costs factor** (MDORS-PC) negatively correlated with visits the owner) during PreBaseline (r = 0.407, *p* = 0.029), interaction with the owner and (r = 0.392, *p* = 0.048) and visit the owner (r = 0.391, *p* = 0.048) during PostBaseline and fear behaviors during DollCar (r = −0.371, *p* = 0.048). Other correlations between MDORS factors and behaviors were statistically non-significant (Table S3).

#### 3.3.4. Correlations of Dog–Owner Relationship (MDORS) with Owners’ Ratings of Dog Emotional State and Dog Personality

Owners’ arousal and valence assessments did not correlate statistically significantly with the factors of dog–owner relationship (MDORS-EC, MDORS-SA, MDORS-PC).

The personality traits of the dogs correlated with the factors of the dog–owner relationship. MDORS-PC correlated positively to self-assuredness (r = 0.458, *p* = 0.012), negatively to trainability (r = −0.553, *p*= 0.002) and negatively to MDORS-EC (r = −0.370 *p* = 0.048). MDORS-EC and MDORS-SA correlated positively (r = 0.437, *p* = 0.018). In addition, extraversion and neuroticism correlated positively (r = 0.435, *p* = 0.018).

Correlations between dog personality traits and behaviors during each test phase are reported in Table S4.

## 4. Discussion

In this study we explored how the dog–owner relationship affects emotional reactivity of dogs, measured from dog physiology (HRV), behavior and owners’ evaluations. This novel multi-method approach revealed that emotional reactions of dogs were mediated by the dog–owner relationship. Owners’ subjective interpretation of dog emotional arousal aligned with HRV and behavioral measures, and was not altered by the quality of the relationship.

**Dog HRV and physical activity in different emotional situations.** Heart rate variability and physical activity both varied across the test phases. Dogs were more active during most of the emotional stimulations in comparison to the baselines. Higher physical activity was correlated with lower HRV, but changes in HRV were not merely due to changes in activity but also dogs’ emotional arousal: when the activity level was taken into account in HRV analysis as a corrective factor, situation dependent HRV changes attenuated, but did not vanish.

Scarce reports exist regarding the effect of physical activity on canine HRV during emotion provoking situation. Maros et al. [10] and Travain [16] recorded HRV during short emotion provoking situations and found that HRV (measured as RMSSD and SDNN, i.e., standard deviation of NN intervals) was not affected by locomotion. In contrast, Ortmeyer et al. [36] measured 24 h/d everyday life activity by PetPaceTM smart collar with inbuilt accelerometer and found that activity level predicted dogs’ HRV measured as vasovagal tonus index. Differences between the studies may be due to methodology: Maros et al. [10] and Travain [16] measured activity level by encoding behaviors (e.g., walking, jumping) from video recordings, which is a much rougher measure than activity tracking by accelerometer that detects also the changes in body position, shaking, etc. [69].

In our study, HRV decreased most strikingly during the Kong phase, where dogs manipulated a food-stuffed toy. Such a stimulus has not previously been used in canine HRV studies, but decrease in HRV has been reported during the anticipation of food or eating desired food [11,12,13]. As the physical activity level was only moderate and not correlated to heart rate variability during the Kong phase, lower HRV likely reflected emotional arousal due to positive excitement and pleasure. In some reports, low HRV has been considered as an indicator for a negative emotional state [15,17] and high HRV for a positive emotional state [20]. However, a change in HRV does not unequivocally tell whether the emotion was negative or positive, but reflects the level of vigilance (i.e., alertness, excitement). The results of the current study remind that also positive emotional state may evoke arousal visible as decrease in HRV. The affective changes in HRV should be interpreted by taking both the potential emotional valence of the stimulation and vigilance level of the dog into account instead of merely one of these.

In primary analysis, the decrease in HRV was observed also during DollCar phase (compared to Stroking). During this situation, the lower RMSSD was correlated with dogs’ fear behaviors and higher activity. Correspondingly, Gácsi et al. [14] found that a frightening situation led to reduced HRV accompanied with clear fear signals. However, in our study, the decrease in HRV during the DollCar tailed off when the activity level was taken into account in the analysis, suggesting that the lowered HRV found in the primary analysis was influenced by physical activity.

Physical activity and emotions are closely intertwined. Emotional reaction can cause changes in HRV both due to emotional processes and behavioral changes. HRV is strongly influenced by those behaviors that are related to locomotion [8]. Usually, fear responses evoke intense locomotion in dogs, for example defensive attacking toward the threatening object and dodging away from it, as was frequently observed during the DollCar phase. This may partially explain the high positive correlation between HRV and activity during DollCar. It is also possible that the changes in RMSSD were at least partially due to emotional responses as movement may cloud the regulation linked to emotional processes [9]. However, it is impossible to disentangle which part of the HRV variation was due to locomotion [9,32]. Therefore, strictly excluding all HRV variation linked to movement may cause false negative findings. 

In our study, the different emotional phases were conducted always in the same order, which may have induced a carryover effect from one phase into another: a previous affective experience may have either strengthened or weakened the following emotional response. For example, Mariti et al. [72] found that stroking by the owner before a separation seemed to have a calming effect during the separation. We cannot rule out some kind of an order effect between the phases, but on the other hand, the situation is the same in any studies with separation–reunion, as these situations cannot be performed in randomized order. Nevertheless, from Figure 2, one can see that HRV, activity and owners’ assessment about the emotional state of the dog fall into the same levels in PreBaseline and PostBaseline, which suggests that there was enough time in our design for the recovery. In future studies, for example, a minute-to-minute moving average might bring more detailed information of the time dependent HRV changes within the emotional situation and after that.

**Links between dog–owner relationship and HRV.** Previously, the dog–owner relationship has been linked to variation in the dog’s cortisol levels [57,61,73]. Here, the quality of a dog–owner relationship was reflected in the dog’s heart rate variability. The three aspects of the relationship [63] were associated with RMSSD differently: the higher emotional closeness (MDORS-EC) and higher perceived costs (MDORS-PC) owners reported, the higher in the RMSSD was, while higher shared activities (MDORS-SA) reported by the owners showed a trend toward lower RMSSD during the experiment. Findings emphasize that while assessing the influence of dog–owner relationship on dogs’ emotional regulation, the different aspects of the relationship should be considered separately instead of, for example, using a total MDORS score.

Emotional closeness (MDORS-EC), the aspect which measures how strong emotional bond owner feels toward the dog [63], was related to increase in the heart rate variability—the stronger the bond, the higher the RMSSD. The increase in RMSSD reflects decrease in sympathetic nervous system activation and increase in parasympathetic activation [8,9], and have been previously associated with a more relaxed affective state in dogs [16,18,19,20,21]. The result supports the theory that attachment relationship to humans has stress-alleviating effect on the dog: in stressful situations, dogs seek assurance and comfort from their owners in the same way as human children use their caretakers as a safety haven [14]. The comforting effect of the owner appears stronger in dog–owner dyads with high MDORS-EC [74]. This phenomenon may be mediated by hormone oxytocin, which promotes emotional bonding and counteracts stress hormone cortisol. Interaction between dogs and the owner stimulates secretion of oxytocin in both parties (reviewed in [49]). In dog–owner dyads with a strong emotional bond, oxytocin levels appear higher and cortisol levels lower than in dyads with weaker bond [57,73]. In our study, dogs with high scores in MDORS-EC exhibited more fear-related behaviors during a Dollcar situation. Responses to such a novel object reflect dogs’ general fearfulness and anxiety [75]. Fearfulness of the dog may facilitate emotional closeness, probably because dogs that are more fearful initiate contact with their owner more often [76].

In human attachment relationships, the caregiver can be seen to provide a secure base for the child, which alleviates the anxiety of the child in novel situations [54]. Similarly in dogs, secure attachment enhances independence of dogs in novel and challenging situations [51,52,55], which appears for example as enhanced exploration of a novel environment and persistence in object manipulation tasks [56]. In the current study, dogs whose owners reported high MDORS-EC stayed longer close to their owners during manipulation of a feeding toy (KONG^®^). Furthermore, the longer dogs kept closer proximity, the longer they interacted with the toy (see Table S2), suggesting that closely bonded owners served a secure base for the dogs. In previous reports, secure base effect has not been directly linked with emotional closeness, but some related findings exist. For instance, during veterinary visits, which are usually distressing situations for dogs, strong emotional owner–dog bond seems to ease hesitation—dogs are more willing to play and take treats [74].

The second aspect of dog–owner relationship, shared activities (MDORS-SA), measures how often the owner interacts with the dog in an affectionate way in their daily life, considering the dogs like a family member (e.g., gives treats and gifts, plays with the dog, hugs and kisses the dog, watches tv with the dog) [63]. This factor appeared to be linked to increased arousal and attachment behaviors of dogs. According to a statistically non-significant trend, the more of shared activities owners reported, the lower heart rate variability their dogs tended to have during the whole experiment. In addition, the behaviors indicating arousal and attention/proximity seeking emerged during certain experimental situations. During PreBaseline, dogs with higher MDORS-SA wagged their tails more and interacted longer with their owners. During Separation higher MDORS-SA correlated with longer duration spent close to the door and during Reunion more self-directed behaviors, yawning and panting. These kinds of arousal and proximity seeking during separation–reunion are typical indicators of attachment bond: they may reflect both affiliation and the distress caused by separation [77].

Rehn et al. [78] found that higher MDORS-SA factor was associated with higher proximity seeking during reunion. This was suggested to be due to dogs’ insecurity or the reinforcing effect of frequent positive interactions between the owner and the dog. It is likely possible that both of these influence each other. Proximity seeking in dogs can be related by both sociability/affiliation and insecurity/stress alleviation [62,79]. Existing evidence shows that owners report more shared activities with dogs that are likely to be more stress susceptible, for example, dogs scoring higher in neuroticism [61] or have high basal cortisol levels [57]. These type of dogs may act the way (i.e., show stress behaviors, seek attention) that triggers their owners’ caring behaviors. In turn, dogs’ attention seeking behavior may facilitate owners’ attachment to the dog [80]. Owners who report frequent shared activities with their dogs consider their dog as a positive and pleasant companion [73] and are satisfied to their dog–owner relationship [57]. Frequent interaction with the dog is likely to be perceived as a positive experience that enhances the feeling of emotional closeness in owners [81]. Correspondingly, in our study, shared activities correlated positively to emotional closeness reported by the owners.

Interestingly, dogs that have stronger bonds according to the owners yawned more often during reunion (yawning correlated positively with both MDORS-EC and MDORS-SA). In addition, yawning was correlated to lower RMSSD (i.e., higher arousal) and self-directed behaviors, corresponding to findings of Kuhne et al. [15]. Usually, dogs first greeted the owners enthusiastically, then shook and stretched themselves accompanied with yawning. Yawning is commonly considered as an indicator of distress [26,69,82]. During the Reunion phase of the test, dogs may have been anxious of being left alone again. In turn, yawning may bear communicative function as proposed for body shaking; these greeting behaviors may act as reinforcers of the social bond [77]. Indeed, during affiliative interaction with humans yawning has been associated with a positive emotional state and attentive behaviors [17,30,79,83]. These behaviors may be a calming mechanism for dogs, and hence signs of relief rather than stress [26,84,85].

The third factor of dog–owner relationship, the perceived costs (MDORS-PC) measures how much the owner feels that the dog limits her/his life and taking care of the dog is not worth the effort [63]. Here, this aspect was positively correlated with dogs’ heart rate variability: the higher MDORS-PC owners reported, the higher was the dogs’ RMSSD. High MDORS-PC were associated with dog’s lesser interaction with the owner during baselines and lesser fear behaviors during the frightening situation (DollCar). MDORS-PC correlated directly with a dog’s personality traits, unlike the other two aspects of the dog–owner relationship. Higher MDORS-PC was linked to dog’s higher self-assuredness, lower neuroticism and lower training focus. Self-assured dogs are described as independent and insistent [64,86], which may evoke the feeling of weaker responsiveness to training for the owners [61]. As a contrast, dogs with high training focus are co-operative and keep focus on the owner. Such attributes enhance owners’ attachment to the dogs [80], and thus, low trainability may affect oppositely. Actually, the lower the emotional closeness was, the higher the perceived costs were, which is expectable, as MDORS-PC reflects the owner’s dissatisfaction with the dog [87] and in turn, satisfaction goes hand to hand with emotional closeness [73]. 

In our sample, high MDORS-PC was related to dogs’ lower arousal through high HRV, lower fearfulness (higher self-assuredness, lower neuroticism and less fear-related behaviors) and lower human orientation (less interaction with the owner, lower trainability). Previously, high MDORS-PC has been linked to lower cortisol levels in both types of dogs present in our sample, Labrador Retrievers [73] and herding dogs [61]. Therefore, it seems possible that dogs with high MDORS-PC of our sample represent dogs with low basal stress levels, reflected by their generally higher HRV. Such dogs may not have strong need to use their owners for stress alleviation. Besides, high MDORS-PC has been associated also to lower oxytocin levels in dogs [73]. This may reflect dogs’ genetical predisposition for weaker emotional bonding as individual sensitivity for oxytocin modulates dogs’ attachment behaviors toward their caretakers [62]. Noteworthy, perceived cost factor may reflect differential characteristics of the dogs and attitudes of the owners in different populations, e.g., depending on whether the breed has been selected for independent working of cooperative purpose (e.g., [57]).

In general, the links between dog’s personality and HRV has been scarcely studied, mainly related to anxiety and aggression [23,24,25]. In our sample, which consisted of competition and working dogs without specific behavior problems, the personality traits did not directly affect their HRV. Previous reports have shown that the type of the owner relationship and other owner-related factors may be an even more significant modulators of dogs’ emotional regulation than dogs’ personality traits [60,61,88]. However, the individual characteristics of the dog are not inconsequential, but may modulate what kind of relationship develops between the dog and the owner [62]. 

**Owner assessment of dog emotion.** According to previous reports, owners’ assessment of their dogs’ arousal are in line with dogs’ stress behaviors [48,89] and cortisol levels [47]. However, dog owners’ assessments of dog emotion have not been coupled with dogs’ cardiac activity before. Our results show that the dog owners recognized both the negative and positive arousal of their dogs, rating arousal highest during manipulation of a feeding toy (Kong) and during the sudden appearance of a strange object (DollCar).

During negative experimental situations, i.e., Separation and frightening object (DollCar), owners’ impressions of more aroused and negative emotion were correlated to lower RMSSD, increased physical activity, and behaviors that typically indicate fear and stress in dogs [26,70,71]. The occurrence of many of these behaviors also correlated with lower RMSSD. During DollCar, owners’ ratings of high dog arousal and low valence corresponded with increased dog vocalizations and fear behaviors (such as startling, bolting, retreating, crouching, freezing and paw lift). People typically easily recognize these behaviors as signs of fear and defense [71,90]. During the Separation phase, owners’ ratings of high dog arousal and low valence corresponded with dogs exhibiting more panting and vocalizations, the behaviors which are considered by owners to indicate stress [82,91]. During PostBaseline, owners’ arousal assessments correlated with the occurrence of stress- and fear-related behaviors of the dog (body shaking, stretching, scratching and self-grooming). Apparently, some dogs had not fully recovered from the preceding stressful situation.

Contrary to the negative situations, owner’s assessments of dog emotion did not directly correlate with either RMSSD or activity during the positive emotional situations. When dog owners evaluated positive affective states of dogs, activity per se appeared not to be very informative. For estimation of the valence of dog affective state owners may have observed their dogs’ holistically paying attention to the combination of cues which are typically considered as a sings of positive emotions in dogs, for example, the general stance, gross body movements and facial expression [4,37,40,90,92]. Owners’ arousal assessments correlated positively with panting and tail wagging, which in turn were correlated to lower RMSSD in dogs, showing that owners recognized arousal also during positive emotions. The valence of dog emotional state was rated as more pleasant in dogs that wagged their tails more, which is expectable as tail wagging is considered as the most typical sign of positive emotional state of dogs [37,90].

It seems that in frightening and distressing situations, the owners of the dogs in our sample who were experienced in dog training could approximate the arousal state of their dogs. However, the current data are uninformative of how accurate and exact the owners’ assessments were, and how much can be generalized to less devoted dog owners, who may not pay attention to such a wide range of gestures of their dogs [82,89,92]. In addition, human interpretation of dog behavior and emotions can be affected by mental state attribution, empathy and cultural factors [1,2,93,94] as well as gender [38,39,82] and parental status [91]. Our dog owners were all female, and this gender bias is usual in this kind of dog owner studies, e.g., [37,40,89,93]; thus, it is not certain whether the results also apply to men. 

Highly attached owners tend to make more emotional attributions of their dogs than less attached owners [37,38,39]. In this study, owner’s assessments of dog arousal or valence were not affected by the quality of dog–owner relationship. This is somewhat contradictory to a previous finding, where owners with a high level of attachment have rated their dogs’ emotional state as happy more often than those with lower attachment [37]. However, the study of Buckland et al. [37] was an online survey, where the dog owners did not observe their dogs’ behavior in situ but answered more based on their general impressions. As the same behavior may occur both during positive and negative emotional situations [5], the behavior of a dog should always be interpreted in the context in which it appears. Furthermore, dog experience appears to lead to a general positive bias toward dogs [95] that may affect situations not strictly tied with dog behavior. Additionally, Buckland and colleagues [37] had a much larger survey sample than in this study, probably including more variation in the dog–owner relationship as well as the level of owners’ experience, which may affect these findings. The sample of this study may be biased toward experienced owners with positive attitude toward their dogs.

## 5. Conclusions

In conclusion, the emotional reactions of dogs were reflected in the dogs’ heart rate variability, behavior, physical activity and owners’ interpretations. The dog–owner relationship seems to be associated with dogs’ emotional reactivity, as reflected in the HRV and behavior of the dogs. Three aspects of the relationship affected dog’s HRV in different ways, and the relationship may be partially influenced by dogs’ personalities characteristics. The emotional closeness aspect of the relationship appears to be related to lower arousal, possibly related to stress alleviation due to secure base effect. The second aspect, the frequency of owners’ shared activities with the dogs, is likely facilitated by dogs’ attachment behaviors, which in turn may be related to dogs’ arousal tendencies. The third aspect, perceived costs, appears linked to dogs’ personality: more independent dogs may not be easily distressed; thus they may not have a strong need to lean on human support, which in turn may result in their owner’s experience of the emotional bond remaining weaker. Therefore, the characteristics of the dog appear to have indirect effects through the development of dog–owner relationship, even though dogs’ personality traits did not influence dogs’ HRV directly.

Although dog–owner relationship was connected to the dog’s emotional reactivity, the quality of the attachment bond did not bias the owner’s ratings about their dog’s emotional state. Owners’ estimates aligned with the HRV findings and behavioral responses especially during the negative incidences. Anxiety- and fear-evoking situations caused decrease in HRV, which was associated with increase in motor activity and behaviors that typically indicate arousal. It is noteworthy that HRV also decreased during a positive situation, without direct associations to activity, behaviors or owner impressions, indicating that physiological measurement may reveal emotion-related responses not directly detectable from the behavior.

## Figures and Tables

**Table 1:** Detailed description of the experimental phases.

Test Phase	Duration	Expected Emotional Valence	Description
Pre-Baseline	10 min	neutral	The owner and the dog in the testing room, the owner sitting on the chair and filling in questionnaire sheets.
Stroking	5 min	positive	The owner and the dog were in the testing room, the owner stroking the dog on the floor. If the dog was not willing to be stroked, owner did not to restrict the dog’s movements or did not force it to be petted.
Kong	5 min	positive	The owner and the dog were in the testing room. The dog was licking/chewing a rubber toy (Kong^®^) filled with food on the floor. The owner sat on the chair.
Separation	5 min	negative	The owner left the dog alone in the testing room, saying goodbye to the dog as she would do while leaving the dog alone in everyday life.
Reunion	5 min	positive	The owner went back to the testing room and greeted the dog as she would do in everyday life. After greeting, the owner went to sit on the chair and was allowed to stroke the dog if dog was seeking contact.
DollCar	5 min	negative	The owner and the dog were in the testing room. The owner sat on the chair. An unfamiliar moving object referred to as “DollCar” (Figure 1; a remote-controlled car with a doll on top of it; a total size approx. 30 × 42 × 20 cm) suddenly emerged from hiding (cabinet in the shelf) and stopped after moving approx. 1 m. 1 min after the DollCar appeared, the owner was instructed to walk to the shelf and back. After 3 min, the owner was instructed to go to the DollCar and turn it on its side. In the cases where the dog was behaving very fearfully, the owner was instructed to go to the DollCar after 1 min.
Post-Baseline	10 min	neutral	The owner and the dog in the testing room, the dog behaving freely and the owner sitting on the chair and filling in questionnaires.

**Table 2:** The average scores of the MCPQ-R and MDORS factors (scale 1–5) and reliability values (Cr-α) of factors after omitted items.

Factors	Mean (SD), Range	Cr-α	Omitted Item
MCPQR-A	4.91 (0.76), 3.50–6.00	0.624	Relaxed
MCPQR-E	3.94 (0.92), 1.33–5.50	0.863	
MCPQR-N	2.22 (0.85), 1.00–4.33	0.727	Submissive
MCPQR-S	4.34 (0.71) 3.00–6.00	0.701	
MCPQR-T	4.99 (0.70) 3.50–6.00	0.745	
MDORS-PC	1.49 (0.36) 1.00–2.44	0.666	
MDORS-EC	3.26 (0.92) 1.30–4.70	0.909	
MDORS-SA	3.52 (0.48) 2.25–4.13	0.643	How often do you have your dog with you while relaxing, i.e., watching TV?

**Table 3:** Ethogram containing the behaviors included in the analysis.

Behavior	Description
Interacting with owner	Duration of dog is gazing at the owner or sniffing, licking or touching the owner with a muzzle, mouth, jaw or paw. Dog may lean/rub its head/body on the owner’s body.
Visit the owner	Frequency of dog gazing at the owner or sniffing, licking or touching the owner with a muzzle, mouth, jaw or paw. Dog may lean/rub its head/body on the owner’s body.
Close to the owner	Duration of the distance between the dog and the owner is less than 1 m
Retreating from Car ^1^	Duration of dog trying to avoid the DollCar by retreating, withdrawing or turning away from it
Freezing/Paw lift ^1^	Duration of dog freezing in its place: stays immobile. Dog may keep one forepaw in the air (without intending to touch the object with it)
Interacting with Kong	Duration of dog is right next to the Kong toy, sniffing, licking or biting it, or touching it with the paw
Interacting with door	Duration of dog is positioned next to the door or the distance between dog and owner is less than 1 m and dog is gazing at the door or sniffing, biting or scratching the door. Dog may jump against the door.
Startling/Bolting ^1^	Duration of dog getting startled making fast retreating movement, boggling/wincing, bolting away
Crouching ^1^	Duration of dog is in a crouched body position, head and tail low. Dog may stay still or move.
Shaking ^2^	Duration of dog shaking its body making a fast rhythmic rotating movement back and forth around its spine.
Stretching ^2^	Duration of dog pulling part of its body in the opposite direction of the remaining part of the body.
Self-grooming ^2^	Duration of dog licking/biting its fur or skin
Scratching ^2^	Duration of dog moving one hind paw rapidly back and forth against the body
Tail wagging	Duration of dog is wagging its tail, tail can be in any position
Vocalization	Duration of dog is vocalizing by barking, growling, whining or howling
Panting	Duration of dog is panting: mouth open, breathing with short, quick breaths.
Yawning	Duration of dog is yawning: prolonged slow open the mouth, opening it exaggeratedly. Usually dog is lifting its nose and squinting its eyes.

^1^ Fear behaviors: Sum of variables above marked with. ^2^ Self-directed behaviors: Sum of variables above marked with.

**Table 4:** Spearman Rank correlation coefficients (Rs) between dog HRV, activity and behaviors during baselines and five experimental conditions (Stroking, Kong, Separation, Reunion, DollCar). Statistically significant correlations are marked with asterisks (* *p* < 0.05, ** *p* < 0.01, *** *p* < 0.001).

	PreBaseline		Stroking		Kong		Separation		Reunion		DollCar		PostBaseline	
	HRV	Activity	HRV	Activity	HRV	Activity	HRV	Activity	HRV	Activity	HRV	Activity	HRV	Activity
Fear behaviors	−0.09	−0.05	~	~	~	~	~	~	~	~	−0.61 **	0.46 *	−0.102	0.69 **
Vocalization	0.08	0.04	−0.12	0.03	~	0.29	−0.44 *	0.49 **	0.36	0.00	−0.72 **	0.39 *	−0.128	0.41 *
Panting	−0.34	0.14	−0.49 *	0.05	~	~	−0.50 *	0.06	−0.38	0.06	−0.37	0.37 *	−0.108	0.38
Tail Wagging	−0.21	0.25	−0.24	0.39 *	−0.08	0.44 *	−0.28	−0.03	−0.48 *	0.36	−0.31	0.52 **	−0.284	0.31
Yawning	−0.12	0.32	−0.11	−0.18	~	~	−0.07	−0.16	−0.59 **	0.05	−0.22	0.14	−0.345	0.71 **
Self−directed behaviors	−0.14	0.594 **	−0.06	0.33	−0.02	0.12	−0.09	0.35	−0.08	−0.07	−0.13	0.36	−0.376	0.81 **
Close to owner	0.08	−0.02	−0.05	0.07	0.02	0.01	~	~	0.23	0.13	−0.11	0.13	−0.180	−0.07
Interact with owner	−0.52 **	0.47 **	−0.12	−0.09	0.06	0.10	~	~	−0.03	0.22	−0.09	0.23	−0.30	0.72 **
Visit the owner	−0.649 ***	0.52 **	0.09	0.23	0.10	0.09	~	~	−0.18	0.11	−0.20	0.39 *	−0.31	0.70 **
Interact with Kong	~	~	~	~	−0.17	−0.05	~	~	~	~	~	~	~	~
Interact with door	~	~	~	~	~	~	−0.35	−0.37	~	~	~	~	~	~

~ The behavior did not occur during the test phase.

**Table 5:** Spearman Rank correlation coefficients (Rs) between dog HRV, activity and owner’s assessments of dog emotion (arousal and valence) during baselines and five experimental conditions (Stroking, Kong, Separation, Reunion, DollCar). Statistically significant correlations are marked with asterisks (* *p* < 0.05, ** *p* < 0.01).

	PreBaseline		Stroking		Kong		Separation		Reunion		DollCar		PostBaseline	
	HRV	Activity	HRV	Activity	HRV	Activity	HRV	Activity	HRV	Activity	HRV	Activity	HRV	Activity
Arousal	−0.08	0.02	−0.30	0.36	−0.27	0.33	−0.55 **	0.50 **	−0.30	0.07	−0.68 **	0.50 **	−0.06	0.46 *
Valence	0.28	−0.13	0.33	−0.19	−0.01	−0.03	0.48 *	−0.50 **	0.30	0.30	0.48 *	−0.26	0.01	−0.47 *

**Table 6:** Spearman Rank correlation coefficients (Rs) between owner’s assessments of dog emotion (arousal and valence) and behaviors during baselines and five experimental conditions (Stroking, Kong, Separation, Reunion, DollCar). Statistically significant correlations are marked with asterisks (* *p* < 0.05, ** *p* < 0.01).

	PreBaseline		Stroking		Kong		Separation		Reunion		DollCar		PostBaseline	
	Arousal	Valence	Arousal	Valence	Arousal	Valence	Arousal	Valence	Arousal	Valence	Arousal	Valence	Arousal	Valence
Fear behaviors	−0.16	0.22	~	~	~	~	~	~	~	~	0.59 **	−0.72 **	0.31	−0.59 **
Vocalization	0.21	−0.14	0.23	0.06	0.28	−0.14	0.61 **	−0.68 **	0.01	0.01	0.56 **	−0.44 *	0.50 *	−0.34
Panting	0.20	−0.33	0.47 *	−0.20	~	~	0.35	−0.44 *	0.59 **	−0.15	0.27	−0.13	0.32	−0.20
Tail Wagging	−0.09	0.14	0.39	0.23	0.42 *	0.45 *	0.37	−0.29	0.51 **	−0.01	0.18	0.09	0.41 *	−0.15
Yawning	0.24	−0.38 *	0.14	0.03	~	~	−0.13	0.23	−0.10	−0.16	0.20	−0.08	0.51 **	−0.55 **
Self-directed behaviors	−0.12	−0.01	0.42 *	−0.06	−0.39 *	−0.01	−0.04	−0.16	−0.07	0.08	−0.09	0.15	0.59 **	−0.44 *
Close to owner	−0.30	0.23	0.09	0.13	0.28	−0.14	~	~	0.14	0.39 *	0.33	−0.46 *	−0.17	0.47 *
Interact with owner	0.28	−0.23	−0.04	0.23	−0.23	0.22	~	~	0.41 *	−0.07	−0.24	0.11	0.38	−0.47 *
Visit the owner	0.19	−0.19	0.23	−0.37	−0.20	0.18	~	~	−0.10	−0.18	−0.00	−0.07	0.43 *	−0.47 *
Interact with Kong	~	~	~	~	0.27	0.04	~	~	~	~	~	~	~	~
Interact with door	~	~	~	~	~	~	0.10	−0.11	~	~	~	~	~	~

~ The behavior did not occur during the test phase.

## Data Availability

Study data are available upon request.

## Supplementary Materials

The following supporting information can be downloaded at: https://www.mdpi.com/article/10.3390/ani12111338/s1, Table S1: The statistical results (Wilcoxon signed rank test) of pairwise comparisons between the test phases for the dog biometric measures (RMSSD and Activity) and the owner assessments (emotional arousal and valence). The *p*-values are Bonferroni corrected. Table S2: Spearman Rank correlation coefficients (Rs) between behavior variables during the baselines and five experimental conditions (Stroking, Kong, Separation, Reunion, DollCar). Table S3: Spearman Rank correlation coefficients (Rs) between dog–owner relationship (MDORS) factors and behavior variables during the baselines and five experimental conditions (Stroking, Kong, Separation, Reunion, DollCar). Table S4: Spearman Rank correlation coefficients (Rs) between dog personality trait (MCPQ-R) factors and behavior variables during the baselines and five experimental conditions (Stroking, Kong, Separation, Reunion, DollCar).
